# The AMR emergency: multi-sector collaboration and collective global policy action is needed now

**DOI:** 10.1080/16549716.2019.1855831

**Published:** 2020-12-27

**Authors:** Jennifer Stewart Williams, Stig Wall

**Affiliations:** a Department of Epidemiology and Global Health, Umeå University, Umeå, Sweden; b Research Centre for Generational Health and Ageing, Faculty of Health, University of Newcastle, Callaghan, Australia

This Special Issue of Global Health Action showcases original research, critical review, innovative methods and informed commentaries on antimicrobial and antibiotic use and resistance. The compilation of eleven papers underscores complex inter-relationships and drivers of resistance across human and animal health, agriculture, the environment, and industry. A common view is that multi-sector collaboration is urgently needed to tackle antimicrobial resistance.

The scoping review is a comprehensive synthesis of 399 papers on antibiotic resistance and prevention from the peer-reviewed and grey literature (2000–2017) [1]. The findings indicate gaps in surveillance systems, particularly in low – and middle-income countries and an abundance of information on local and national uses and misuses of antibiotics. An epidemiological framework with potential entry points for antibiotic resistance is suggested. New social and behavioural research methods are needed to complement existing biomedical and clinical approaches.

A second review article appraised microbiological evidence on the prevalence of multidrug-, extensive drug-, and pandrug-resistance in escherichia coli isolates from human sources in community settings in low- and middle-income countries [2]. Nkansa-Gyamfi and co-authors call for greater community-level efforts in designing new and improved public health policies to counter the global threat of antibiotic-resistant infections and bacteria.

Three papers used qualitative methods to explore awareness of antimicrobial resistance in low- and middle-income countries. Pearson and Chandler conducted interviews with human and animal healthcare professionals in Ethiopia, India, Nigeria, the Philippines, Sierra Leone and Vietnam [3]. Their finding that awareness of antimicrobial resistance did not translate to reduced prescribing is concerning. Barriers persist in contextual and structural factors such as accessibility and affordability, lack of local antibiotic information, and poor hygiene and sanitation. Contextual factors such as affordability and accessibility are also highlighted in a qualitative study of antibiotic use in rural communities in Bangladesh by Chowdhury et. al. [4]. Cultural and religious beliefs inform medicine use. Advice on antibiotics is typically sourced from unregulated village pharmacies. Lack of trust in government run hospitals and clinics is a sharp contrast. The focus on individual behaviour change needs to take the precarity of individuals’ socioeconomic circumstances into account. In another study conducted in Bangladesh, Darj, Newaz and Zaman conducted in-depth interviews with retail pharmacists in Dhaka to help understand their perceptions regarding antimicrobial resistance [5]. Participants reported self-medication, old prescriptions, inadequate regulation and readily available antibiotics as main contributors.

We have noted a lack of educational and stewardship programmes for antibiotic resistance [1]. Hospital-based antibiotic stewardship programs can improve patient outcomes and lower the risk of antibiotic resistant infections. McKnight et. al. [6] used mixed methods to investigate antibiotic stewardship policies and structures in 16 Kenyan hospitals. National policies should build upon existing structures, responsibilities and practices to manage the supply and prescription of antimicrobials.

Antibiotics are used in high volumes to enhance growth and prevent disease in food producing animals. The Short Communication article by Mohsin et.al [7]. describes the results of an important baseline study which highlights worrying concerns about the use of antimicrobials in the poultry industry in Pakistan. The country’s consumption intensity of 250.84 mg of active ingredient per kilogram of chicken in Pakistan is second only to China’s. In their Short Communication article, Thornber et. al investigated digital communication as a means of rapidly and effectively communicating antimicrobial resistance messages to farmers in rural aquaculture in Bangladesh [8].

Disciplines such as psychology, sociology and anthropology are critical for understanding the developmental, socioeconomic and political contributors to antimicrobial resistance. Methods Forum articles showcase cross-disciplinary tools applied in global health research. The paper by Dixon et.al. on the ‘Drug Bag’ Method demonstrates the use of anthropological methods to investigate antibiotic recognition, use and accessibility [9]. A paper by Haenssgen used standard evaluation criteria from the field of development aid evaluation to promote more systematic and comprehensive evaluation practice on antimicrobial resistance, use and health behaviour in Thailand and Laos [10]. Mitchell et.al. suggest seven values – clarity, creativity, evidence-led, equity, interdisciplinary, sustainability and flexibility – for underpinning community engagement approaches to tackling antimicrobial resistance [11]. Being responsive to community needs and circumstances opens opportunities to build collaborative, community centred, multi-sector approaches to address antimicrobial resistance.

Antimicrobial resistance is a threat to global health and food security. Analogies with climate change include the irreversible damaging consequences of inaction. The emergence of COVID-19 in 2020 has focused attention on yet another global health challenge. The difference is that the pandemic is seen as a proximate public health crisis requiring immediate action. Consequently, relatively less attention has been directed towards antimicrobial resistance and climate change which also pose urgent threats to lives and livelihoods, and in the end, may have worse global consequences. Media reports and scientific literature on COVID-19 have been prolific in 2020. A search in the Web of Science including Medline gave 78,000 ‘hits’ on COVID-19, 38,000 on climate change and 17,000 on antibiotic or antimicrobial resistance. The results from a search in the Swedish Media Archive yielded 323,000, 11,000 and 1,100 respectively, which was even more revealing.

COVID-19, antimicrobial resistance, biodiversity loss and climate change result from human-induced actions that alter the ecological balance and context in which disease hosts evolve. As the climate warms more viruses will be activated and infections will spread. Inappropriate use of medicines to treat viruses and bacteria will further contribute to resistance.

Individually the threats posed by infectious diseases, antimicrobial resistance and climate change are enormous. The synergy between them requires urgent attention and collective global policy action. This is imperative if we are to understand how to minimise losses and achieve equitable and sustainable global health and economic development.

The development of resistance, despite the fact that it threatens our vested right to the best care, reminds us that the poorest in the world are still more affected by the lack of access to regulated antibiotics than by the resistance. Preserving the effectiveness of antibiotics while ensuring universal access is therefore an ethical obligation.
